# Microevolutionary dynamics of eccDNA in Chinese hamster ovary cells grown in fed-batch cultures under control and lactate-stressed conditions

**DOI:** 10.1038/s41598-023-27962-0

**Published:** 2023-01-21

**Authors:** Dylan G. Chitwood, Qinghua Wang, Stephanie R. Klaubert, Kiana Green, Cathy H. Wu, Sarah W. Harcum, Christopher A. Saski

**Affiliations:** 1 Department of Bioengineering, Clemson University, Clemson, SC USA; 2 Center for Bioinformatics and Computational Biology, University of Delaware, Newark, DE USA; 3 Department of Chemical and Biomolecular Engineering, Clemson University, Clemson, SC USA; 4 Department of Biological Sciences, University of South Carolina, Columbia, SC USA; 5 Department of Plant and Environmental Sciences, Clemson University, Clemson, SC USA

**Keywords:** Genome evolution, Mobile elements, Transcriptomics, Genomic instability

## Abstract

Chinese hamster ovary (CHO) cell lines are widely used to manufacture biopharmaceuticals. However, CHO cells are not an optimal expression host due to the intrinsic plasticity of the CHO genome. Genome plasticity can lead to chromosomal rearrangements, transgene exclusion, and phenotypic drift. A poorly understood genomic element of CHO cell line instability is extrachromosomal circular DNA (eccDNA) in gene expression and regulation. EccDNA can facilitate ultra-high gene expression and are found within many eukaryotes including humans, yeast, and plants. EccDNA confers genetic heterogeneity, providing selective advantages to individual cells in response to dynamic environments. In CHO cell cultures, maintaining genetic homogeneity is critical to ensuring consistent productivity and product quality. Understanding eccDNA structure, function, and microevolutionary dynamics under various culture conditions could reveal potential engineering targets for cell line optimization. In this study, eccDNA sequences were investigated at the beginning and end of two-week fed-batch cultures in an ambr^®^250 bioreactor under control and lactate-stressed conditions. This work characterized structure and function of eccDNA in a CHO-K1 clone. Gene annotation identified 1551 unique eccDNA genes including cancer driver genes and genes involved in protein production. Furthermore, RNA-seq data is integrated to identify transcriptionally active eccDNA genes.

## Introduction

Chinese hamster ovary (CHO) cell lines are broadly used in the manufacturing of biopharmaceuticals due to ease of culture, adaptability to manufacturing processes, and tolerance to genetic manipulation^1,2^. While CHO cell lines are immortalized and capable of indefinite culture, the adaptability of CHO cell lines can lead to unintended phenotypic drift, referred to as cell line instability. For example, the most common biopharmaceutical products produced by CHO cells, monoclonal antibodies (mAbs), are metabolically challenging to produce. Exclusion of the transgene is a common mechanism to alleviate the cell’s metabolic burden at the cost of losing productivity^3^. This loss of culture productivity is one of the barriers to continuous biomanufacturing^4^. The most common culture method in biomanufacturing is fed-batch cultures where the bioreactor is periodically supplemented with nutrients; however, these additions contribute to the accumulation of metabolic waste products such as ammonia and lactate, that impart a stressful environment on the cells which can induce genome instability and culture termination^5–7^.

The clonability of CHO cells is partially due to the inherent plasticity of the CHO genome^8^. This plasticity can lead to rearrangements and variant accumulation within critical regions of the genome, such as DNA repair mechanisms, that lead to genome instability^9,10^. Most recombinant CHO cell lines exhibit genome instability after a short time due to the inherent plasticity of the CHO genome^2,4,11^. Genome instability can have multiple detrimental effects on cultures such as decreased productivity, poor product quality, and decreased cell viability^4,12^. Various engineering attempts to maintain genome stability have been explored such as site-directed transgene integration^13^, promoter engineering^14,15^, and waste product reduction^16,17^ with varying levels of success: Site-directed integration allowed for more consistent generation of clones with transgenes inserted into stable safe harbors, modifications of the cytomegalovirus (CMV) promoter prevented reduction of productivity in some clones, and alternative feeding strategies, such as pH-mediated delivery of glucose, reduced the accumulation of lactate.

A poorly understood genomic entity that contributes to gene expression alterations, chromatin maintenance, and genetic heterogeneity that may also have a role in cell line instability and phenotypic drift in CHO cells is extrachromosomal circular DNA (eccDNA). EccDNA is a hallmark of genome plasticity^18–25^ and has been identified within many eukaryotes such as yeast, plants, and drosophila^26–30^. In humans, eccDNA has been observed to contain amplified oncogenes and drug-resistant genes in cancers^31–34^; and in blood plasma^31–33,35^. The broad prevalence of eccDNA across kingdoms, as well as in both diseased and normal tissue^35^, likely indicates a conserved biological function. The eccDNA content in an organism seems to be dynamic and change as cells age in terms of abundance, size, sequence composition, and structural peculiarities^22,23,36,37^. These circularized, focal amplifications of small segmental chromosomal DNA look and function similar to episomes and constitute a rapidly accessible pool of genetic heterogeneity for the cell to utilize as the environment changes^37^. EccDNA are often found in high copy numbers, which can impart ultra-high levels of gene expression^28,33,38^. Gene overexpression can serve as a rapid stress response mechanism^39^, which could lead to genetic mosaicism and phenotypic drift^32^. Historically, eccDNA were first observed in CHO cells by Stanfield et al. in 1984 where they reported the presence of circular DNA with high homology to repetitive sequences^40^. Further sequencing studies confirmed eccDNA are partially composed of satellite DNA and show evidence of homologous recombination during biogenesis^41^; however, neither study identified genes encoded on eccDNA in CHO cells.

This study aims to characterize the sequence structure, function, and microevolutionary dynamics of eccDNA within a monoclonal antibody-producing CHO cell line grown in tightly controlled fed-batch cultures. Samples were collected at the beginning and end of cultures for sequencing. A lactate stress was added to duplicate bioreactors to understand the impact of culture stress on eccDNAs. EccDNAs were discovered and annotated for genes and structural features such as repeat motifs, transfer RNA (tRNA) content, and replication origins. The identified genes were mapped to the respective human orthologs for functional profiling in gene ontology (GO) and KEGG pathway analyses. Transcriptome data was also obtained and intersected with eccDNA data to identify potentially transcriptionally active eccDNA genes. Characterizing the dynamics of eccDNA content, or the circulome, in recombinant CHO cells under control and lactate-stressed conditions will improve our understanding of genome plasticity, cell line instability, stress response mechanisms, and implications in biopharmaceutical manufacturing.

## Materials and methods

### Cell culture

Clone A11, a recombinant CHO-K1 cell line expressing an anti-HIV monoclonal antibody (VRC01) that was generated and donated by the NIH, was scaled up through four passages at three-day intervals post thaw from 1 mL working cell banks stored in liquid nitrogen. The inoculum train was expanded in 250 mL shake flasks with a 70 mL working volume and maintained at 37 °C, 5% CO_2_, and 180 rpm. Bioreactors used in this study were ambr^®^250 vessels (Sartorius Stedim, Gottingen, Germany) with two pitched blade impellers and an open pipe sparger (vessel part number: 001-5G25). Bioreactors were inoculated with a target seeding density of 0.4 × 10^6^ cells/mL and a working volume of 210 mL in ActiPro media (Cytiva) supplemented with 6 mM of glutamine. Feeding began on Day 3 and followed a pyramid feeding scheme (3%/0.3% v/v Days 3–4, 4%/0.4% v/v Days 5–6, 5%/0.5% v/v Days 7–8, 4%/0.4% v/v Days 9–10, 3%/0.3% v/v Day 11 and beyond) with Cell Boost 7a/b (Cytiva), respectively.

Temperature and pH were controlled to 36.5 °C and 6.9 + /- 0.1, respectively. The pH was maintained using CO_2_ and sodium bicarbonate; dissolved oxygen (DO) was maintained at 50%. The PID settings have been previously reported as the results of the third tuning in Harcum et al.^42^. A 10% antifoam solution (Cytiva) was added via a control loop as needed. To induce a lactate stress, a highly concentrated (1.338 M) sodium lactate solution was added at 12, 24, and 36 h post-inoculation to duplicate cultures to increase the lactate concentration in 10 mM increments for a total 30 mM addition. Bioreactors were sampled daily for cell density (Vi-Cell, Beckman Coulter), metabolite concentrations (Cedex Bio Analyzer, Roche), and to collect cell pellets for eccDNA and RNA analysis. Cell pellets were obtained by centrifuging culture broth at approximately 10,000 × g for 10 min at 4 °C, treated with RNAlater, and stored at − 20 °C until needed for nucleic acid extraction.

### Library preparation

Cell pellets were split for RNA and gDNA extraction. Extractions were conducted with RNeasy midi kits (Qiagen, 74004) and DNeasy Blood and Tissue kits (Qiagen, 69504), respectively, per the manufacturer’s instructions. Extracted RNA was quantified using a NanoDrop spectrophotometer and treated with DNase before sequencing. The gDNA was quantified using a Qubit Fluorometer (Thermo) prior to circular DNA enrichment. EccDNA was randomly amplified per the Circular DNA Enrichment sequencing (CIDER-Seq) protocol^43^. CIDER-Seq uses a Phi29 polymerase and exo-resistant random primers to randomly amplify circular DNA via rolling-circle amplification. This reaction was performed at room temperature over an 18-h incubation. After amplification, circular sequences were debranched and the branches released and repaired to improve yield. EccDNA was then isolated using magnetic bead purification (KAPA Pure Beads, Roche, KK8000) prior to sequencing. SMRTbell barcodes were adapted to the samples by the sequencing vendor prior to sequencing using a PacBio Sequel II with HiFi reads.

### Bioinformatic pipeline

The DeConcat algorithm was used to process the raw sequence data obtained from PacBio Sequel sequencing^43^. The confirmed eccDNA sequences for each replicate were compiled into a singular file per experimental condition and clustered to a 90% similarity threshold using CD-HIT^44,45^. The clustered sequences were then screened for repeat sequences using RepeatMasker v4.1.1 (Smit, AFA, Hubley, R & Green, P. *RepeatMasker Open-4.0*.2013–2015 < http://www.repeatmasker.org >) to characterize and mask repetitive motifs before annotating gene content using Maker^46^*.* The confirmed eccDNA sequences were mapped to respective chromosomal origins and intersected with 500 kbp genome windows to characterize biogenesis locations^47,48^. The content of tRNA was summarized using tRNAscan-SE 2.0^49^. Functional profiling of eccDNA genes was conducted using gene ontology (GO) and KEGG pathway analyses (detailed below)^50^ with ClusterProfiler^51,52^. Furthermore, to annotate potential origins of replication, databases of known mammalian origins of replication and autonomous replication motifs were compiled from NCBI (retrieved on 07/14/2022, Supplementary Table S1) and used to BLAST search against a confirmed eccDNA sequence database^53^. The raw RNA-seq data were cleaned of sequencing adapters and low-quality bases with the Trimmomatic^54^ software and quality checked with FastQC, respectively. Clean sequence data was aligned to the reference transcriptome using Bowtie2 read aligner^55^, transcript abundance calculated using RSEM^56^, and differentially expressed genes identified using edgeR^57^ (*p* < 0.001, FDR < 0.05). EccDNA and RNA sequences were called for variants against the Chinese hamster PICRH and CHO-K1 reference genomes using Varscan^58^. Transcripts containing SNPs that suggest an eccDNA template were then visually analyzed using Integrative Genome Viewer (IGV)^59^.

### Gene function analysis and literature mining for eccDNA and genome instability-linked genes

Human eccDNA-relevant genes from literature were identified by Entrez Gene IDs from PubTator gene annotations^60^. PubMed Medline was queried with “extrachromosomal DNA” (ecDNA) and “extrachromosomal circular DNA” (eccDNA) to obtain available full-text article PMCIDs. The PMCIDs were used to retrieve the BioC xml files accessible from PubTator Central, which provides gene annotations in the full text articles ^61^. The tool ezTag^62^ was used to display the BioC xml files to allow for efficient manual curation of the gene entities (namely, to remove the non-relevant genes mentioned only in the reference sessions, and validate gene annotations provided by PubTator). The curated eccDNA-relevant human genes (N = 431) were collected and hereinafter referred as eccDNA-relevant genes known from literature (Supplementary Table S2). Chinese hamster genes with human orthologs were identified based on NCBI ortholog assignment (ftp://ftp.ncbi.nlm.nih.gov/gene/DATA/gene_orthologs.gz release of 02/23/2022, Supplementary Table S3). ClusterProfiler was used for GO and KEGG pathway enrichment analysis^52^. Human genes linked to genomic instability (N = 2897) from literature^7^ were also used to intersect with the genes on CHO eccDNAs detected in this study.

## Results

### Phenotypic cell culture data

**Figure 1:**
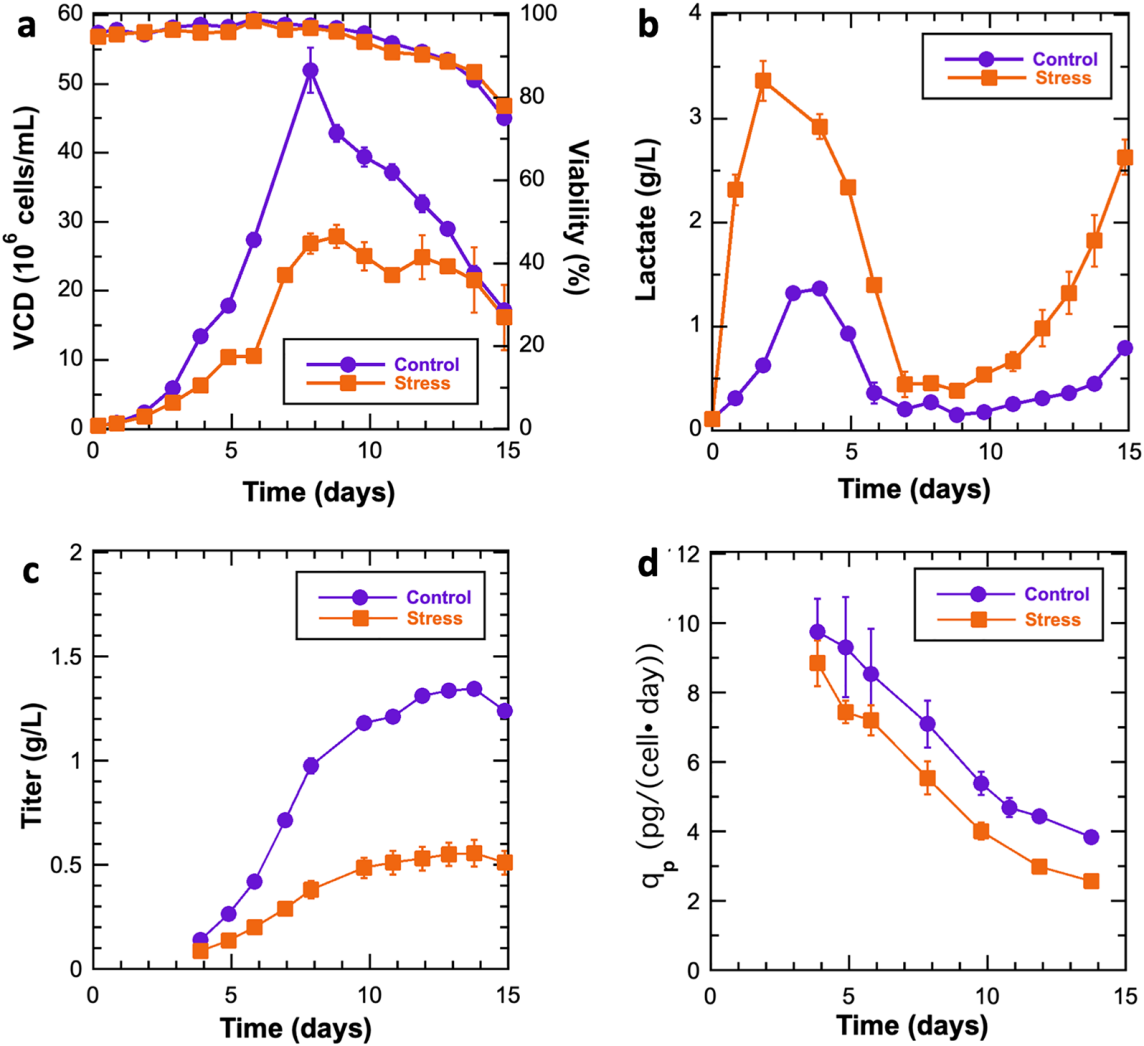
Growth characteristics for control and lactate-stressed CHO cell cultures. (**a**) Viable cell density (VCD) (10^6^ cells/mL) and cell viability (%), (**b**) Lactate (g/L), (**c**) Titer (g/L), and (**d**) Cell-specific productivity (pg/cell•day). Control cultures (purple circles) and stressed cultures (orange squares) N = 2. Error bars represent standard deviations.

To characterize eccDNA dynamics within CHO, cells expressing VRC01 were cultured in an ambr^®^250 bioreactor system under fed-batch conditions in duplicate cultures with and without a lactate stress. Samples for eccDNA analysis were taken immediately after cells were inoculated into the bioreactor (Day 0), which resulted in four replicate samples (N = 4). The duplicate control and duplicate lactate-stressed samples for eccDNA analysis were collected on Day 12 (Control Day 12, Lactate-stressed Day 12 respectively; N = 2 each). The lactate stress resulted in lower growth rates, yet did not negatively impact cell viability (Fig. 1a). Lactate-stressed cultures began lactate consumption on Day 2, while control cultures switched to lactate consumption on Day 4 (Fig. 1b). Recombinant protein titers were lower for the lactate-stressed cultures (Fig. 1c). The cell specific productivity was also lower for lactate-stressed cultures (Fig. 1d).

### Characterization of eccDNA sequence structure and gene content

EccDNAs were captured, sequenced, and verified following the CIDER-Seq pipeline (See Methods). The eccDNA predict algorithm identified 95,517 sequences across the three experimental conditions. Clustering of the eccDNAs at a similarity threshold of 90% collapsed similar sequences together to account for sequencing errors and short reads to a total of 76,317. Sequence length ranged from 21 to 24,309 bp. Mean sequence length was 4063 bp in Day 0 samples and was a bit lower for the Day 12 samples, 3579 bp and 3534 bp for control and lactate-stressed Day 12 samples, respectively. Approximately 37% of bases were identified as repetitive motifs and masked. Long interspaced nuclear elements (LINEs) were the most abundant repeats identified in all three experimental groups followed by long terminal repeat (LTR) elements and short interspaced nuclear elements (SINEs). Distribution of repetitive motifs were mostly consistent across conditions, though lactate-stressed Day 12 samples had more LINEs (16.0%) than control Day 12 (14.3%) or Day 0 (13.3%) samples. tRNA motifs were predicted and found to be in relatively high abundance among the observed eccDNAs. For the Day 0 samples, there were 4520 sequences (9.81%) that contained one or more tRNA motifs while the Control Day 12 and Lactate-stressed Day 12 samples had 1182 (7.86%) and 1151 (7.56%) tRNA motifs, respectively. The full tRNA annotation data can be found in Supplementary Tables S4–6. A database of known mammalian origins of replication was queried to identify sequences harboring motifs associated with autonomous replication. In the Day 0 samples, 4639 sequences (10.1%) were observed to have an origin of replication motif with 95% or greater homology to a known mammalian origin of replication. For the Day 12 samples, only 134 (0.89%) and 12 (0.078%) origin of replication motifs were found in the Control and Lactate-stressed samples, respectively. The full origin of replication results can be found in Supplementary Tables S7–9 and a detailed summary of sequence composition can be found in Table 1.

**Table 1:** Sequence characteristics for eccDNA sequences from control and lactate-stressed cultures including repetitive motif content, gene content, tRNA content, and potential origins of replication.

Condition	Sequences	Sequences clustered	Max Length (bp)	Average Length (bp)	Total bases	Repeat bases masked	GC (%)	eccDNA with genes	eccDNA with tRNA	ORI (> 95%)
Day 0	56,460	46,053	24,113	4063	187,135,316 bp	67,898,424 bp (36.28%)	40.85%	1622 (3.52%)	4520 (9.81%)	4639
Control day 12	19,354	15,047	24,309	3579	53,852,662 bp	19,774,911 (36.72%)	40.92%	486 (3.23%)	1182 (7.86%)	134
Stressed day 12	19,703	15,217	21,482	3534	53,776,705 bp	20,494,955 bp (38.11%)	40.51%	451 (2.96%)	1151 (7.56%)	12

		Day 0			Control day 12			Lactate-stressed day 12		
Repeat structure	Subcategory	Number of sequences	Base pairs (bp)	Percent of total bases	Number of Sequences	Base pairs (bp)	Percent of total bases	Number of Sequences	Base pairs (bp)	Percent of total bases
SINEs:		117,690	15,191,341	8.12	31,575	4,032,279	7.49	30,448	3,883,277	7.22
	Alu/B1	49,213	5,792,592	3.10	13,346	1,563,504	2.9	12,844	1,497,972	2.79
	MIRs	6756	759,480	0.41	1764	195,634	0.36	1643	186,465	0.35
LINEs:		57,169	24,875,542	13.30	16,901	7,686,694	14.3	18,410	8,590,588	16
	LINE1	52,788	562,110	12.90	15,764	7,508,682	13.9	17,240	8,407,560	15.6
	LINE2	3575	83,775	0.3	918	144,521	0.27	932	149,331	0.28
	L3/CR1	567	83,775	0.04	160	24,152	0.04	166	23,420	0.04
	RTE	213	34,675	0.02	54	8805	0.02	63	9279	0.02
LTR elements:		62,652	17,620,142	9.42	18,266	5,017,165	9.32	18,007	4,909,086	9.13
	ERVL	5964	1,634,524	0.87	1700	461,014	0.86	1650	420,104	0.78
	ERVL-MaLRs	28,941	7,395,572	3.95	8234	2,054,567	3.82	8092	1,991,508	3.7
	ERR_class I	5591	1,136,525	0.61	1709	330,819	0.61	1645	326,866	0.61
	ERV_class II	21,634	7,209,443	3.85	6450	2,089,133	3.88	6451	2,103,571	3.91
DNA elements:		11,960	2,185,216	1.17	3351	588,742	1.09	3250	567,968	1.06
	hAT-Charlie	7181	1,238,630	0.66	2060	340,327	0.63	2002	329,155	0.61
	TcMar-Tigger	3066	635,180	0.34	808	161,611	0.3	803	162,637	0.3
Unclassified:		2445	921,187	0.49	684	255,392	0.47	768	322,187	0.6
Total interspaced repeats:		–	60,793,428	32.5	–	17.580,272	32.7	–	18,273,106	34
Small RNA:		3405	267,780	0.14	1071	87,090	0.16	929	71,975	0.13
Satellites		6319	2,860,952	1.53	1994	937,053	1.74	2066	1,001,624	1.86
Simple repeats		74,529	3,580,819	1.91	21,490	1,054,443	1.96	20,814	1,033,385	1.92
Low complexity		9021	456,155	0.24	2547	130,081	0.24	2518	130,232	0.24

Sequences were grouped by condition and clustered for similarity (> 90%). The Day 0 samples include all four bioreactors, while Day 12 samples include duplicate bioreactors for control and lactate-stressed respectively.

**Figure 2:**
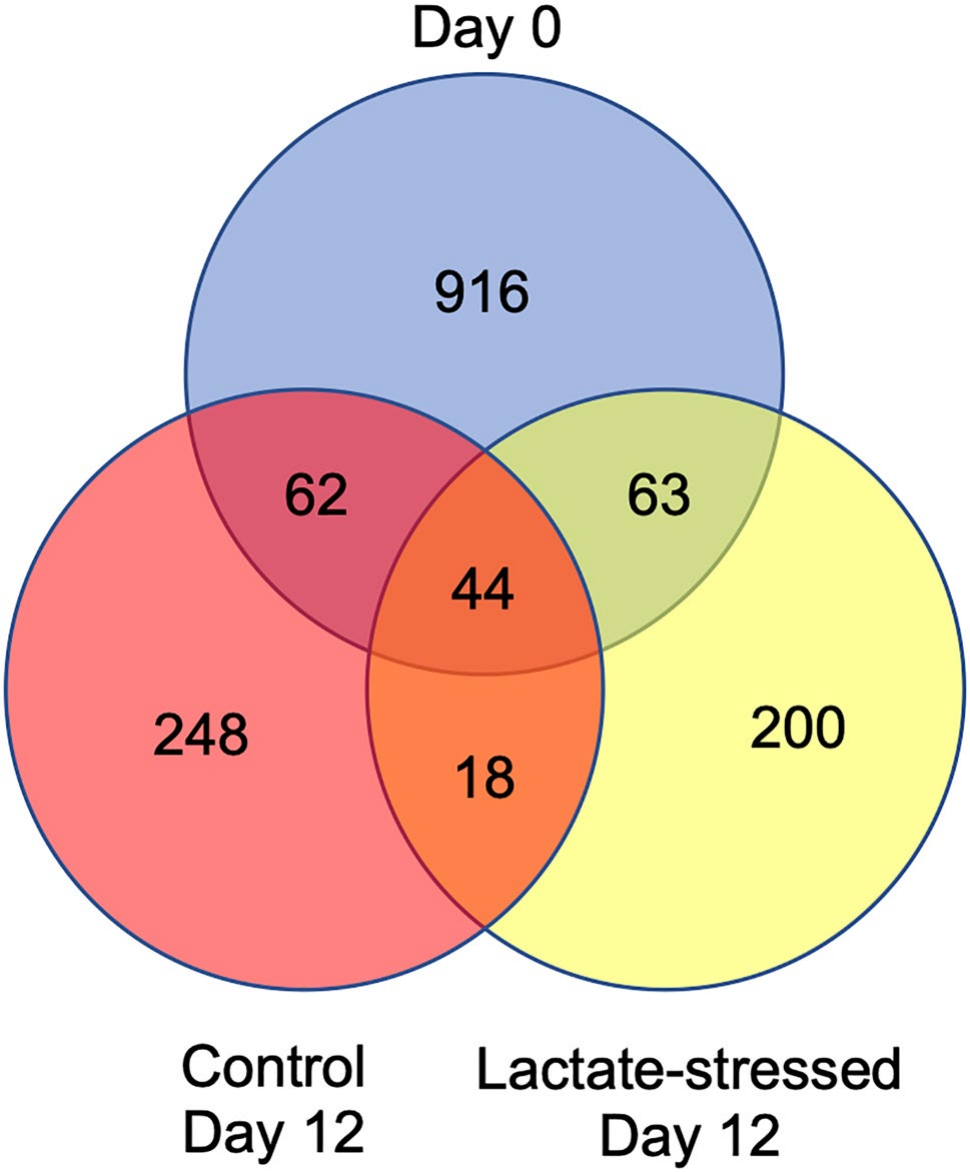
Venn diagram of the genes identified on eccDNA using CIDER-Seq. Day 0 is blue, Control Day 12 is red, and Lactate-stressed Day 12 is yellow.

Next, the 76,317 eccDNAs were analyzed for coding sequences (protein-coding genes). A total of 2559 sequences (3.35%) were found to harbor one or more genes or gene fragments. All gene annotations are listed in Supplementary Tables S10–12. The distribution of gene content was observed to be relatively consistent across the three conditions (3.52% of Day 0, 3.23% of Control Day 12, and 2.96% of Lactate-stressed Day 12 sequences). These sequences contained 1551 unique genes across the three conditions. The majority of these genes, 1364 (88.0%), were only observed in one of the three conditions. However, 143 genes (9.21%) were observed in two conditions and 44 genes (2.83%) were observed in all three conditions. Interestingly, the gene content distribution was biased towards the Day 0 samples which included four bioreactors versus only two bioreactors for the control and lactate-stressed cultures, as the Day 0 samples were biologically identical at this timepoint. The distribution of genes across the conditions is summarized in Fig. 2.

### EccDNA-encoded gene functional enrichment and text-mining analysis

**Figure 3:**
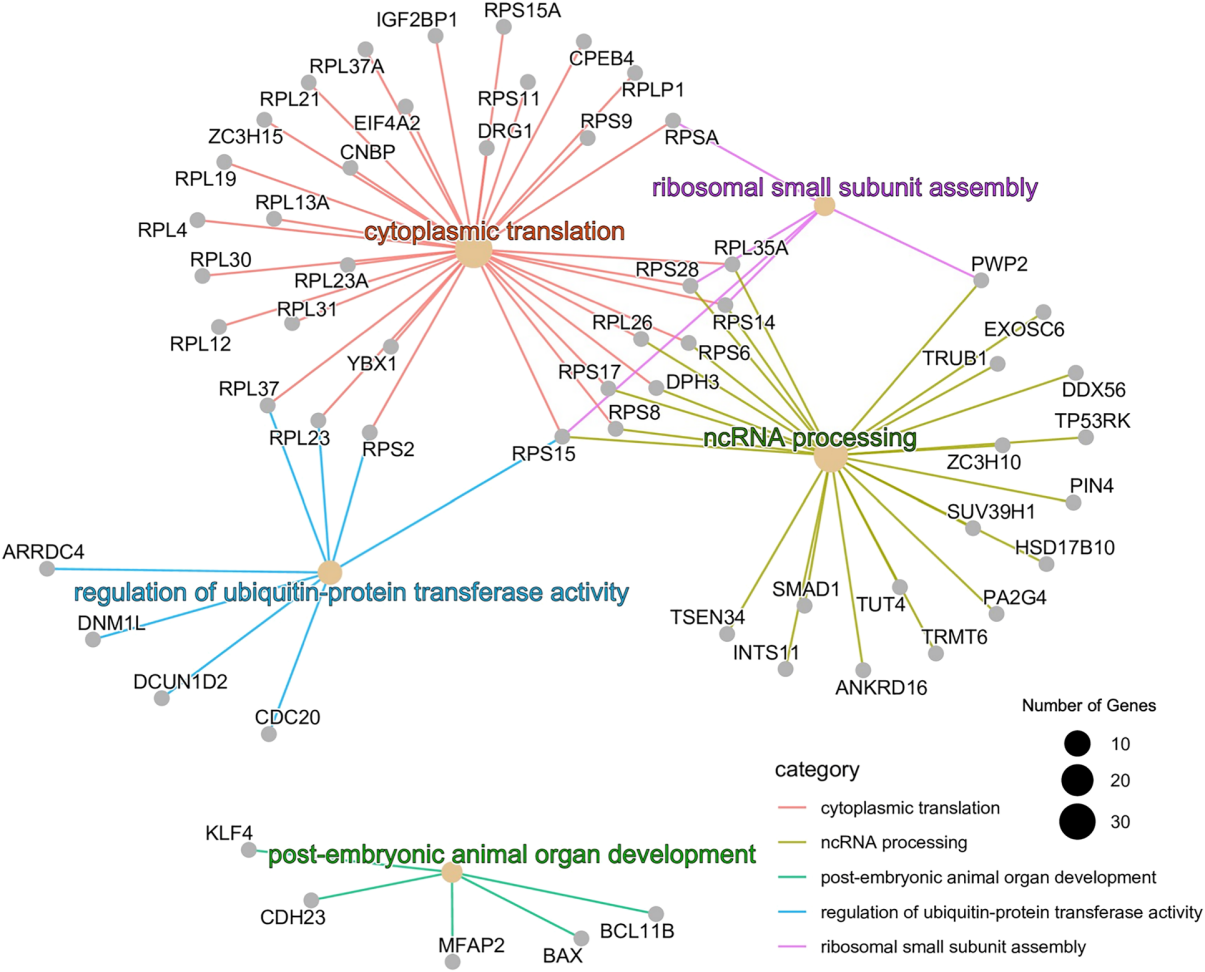
Network diagram of significantly enriched GO biological process terms (adjusted *p*-value < 0.1) for the human orthologs of Chinese hamster genes detected in Day 0 samples. The small gray nodes show individual genes and larger beige nodes indicate GO terms. The size of the beige nodes is proportional to the number of genes with that GO term and the colored lines indicate the GO category for which a gene belongs to.

A survey of biological function of the detected genes in CHO culture eccDNA sequences was performed using the enrichment analysis of GO hierarchy^63^ and KEGG pathways. Gene lists for each culture condition were analyzed for enriched functions. Multiple GO biological process terms were found to pertain to translation, such as cytoplasmic translation, non-coding RNA (ncRNA) processing, and ribosome assembly. A network plot for the Day 0 GO biological process terms is shown in Fig. 3; network diagram for GO terms observed in Lactate-stressed and Control samples on Day 12 are shown in Supplementary Figs. S1 and S2. KEGG pathway analysis also showed significant enrichment in Ribosome and Coronavirus Disease COVID-19 pathways; Lactate-stressed Day 12 samples also had significant enrichment in the Folate biosynthesis pathway (Supplementary Fig. S3).

**Figure 4:**
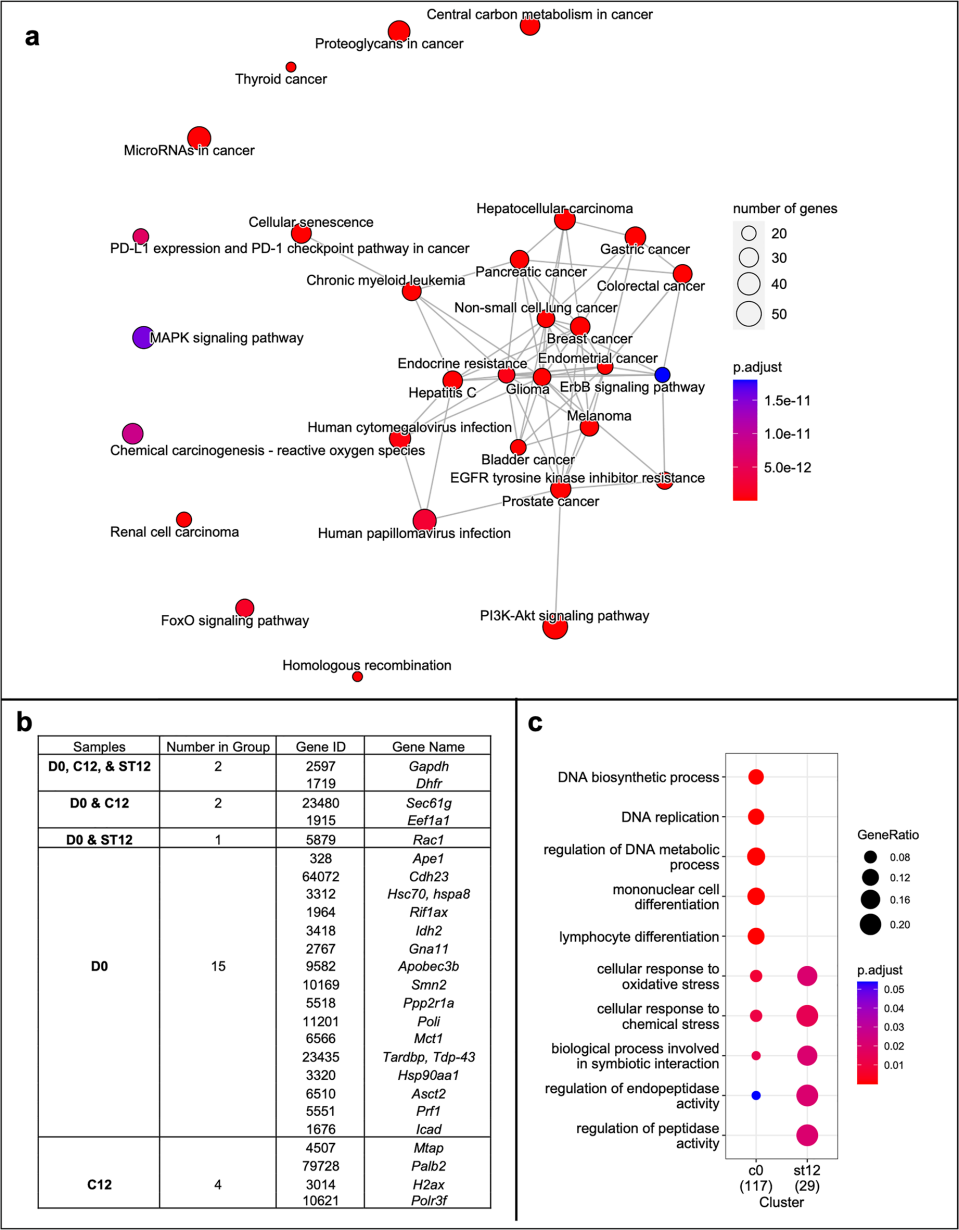
Summary of literature mining results for eccDNA-relevant genes and genome instability linked genes known from literature: KEGG pathway enrichment and CHO culture eccDNA genes. (**a**) Top 30 of the 151 enriched KEGG pathways for the eccDNA-relevant genes texted mined from literature. The node size is proportional to the number of genes found in the pathway, while the node color indicates the pathway’s statistical significance. Pathways with significantly overlapping genes are connected by grey lines. (**b**) EccDNA-relevant genes identified via literature mining that were also found in CHO culture eccDNA sequences from one or more culture conditions: Day 0 (D0), Control Day 12 (C12), Lactate-stressed Day 12 (ST12). (**c**) GO biological process enrichment analysis of genome instability linked genes observed on CHO cell eccDNA. Gene ratio reflects the fraction of genes pertaining to a GO term and the color indicates the statistical significance.

A total of 566 unique genes were manually curated from eccDNA-relevant literature available from PubTator Central (see Methods), which included 431 human genes. For these eccDNA-relevant human genes from literature, the enrichment analysis found 151 significantly enriched KEGG pathways (*p*-value < 0.05) (Supplementary Table S13). Notably, many of the enriched pathways pertained to cancer (Fig. 4a). Multiple eccDNA-relevant genes associated with cancer progression in humans were also observed on eccDNA sequences in CHO cells (Fig. 4b). Several cancer driver genes are amplified via eccDNA-mediated gene duplications in various tumor types^34^. Specifically, *Rac1* was observed in both the Lactate-stressed Day 12 and Day 0 samples; *Eef1a1* was observed in Day 0 and Control Day 12 samples; *Eif1ax*, *Gna11*, *Idh2* and *Ppp2r1a* were observed in Day 0 samples. Additionally, 2 genes were identified in all three conditions: *Gapdh* (glyceraldehyde-3-phosphate dehydrogenase) and *Dhfr* (dihydrofolate reductase). The presence of *Dhfr* on eccDNA sequences in CHO is notable as it is a common selectable marker gene used in CHO cell line development and was the selectable marker for the clone used in this study^64^. Further, CHO eccDNA genes were queried for relation to genome instability genes identified via literature mining, and 117 genes in the Day 0, 31 genes in the Control Day 12, and 29 genes in the Lactate-stressed Day 12 samples were identified (Supplementary Table S14). Functional profiling of genes associated with genome instability revealed significantly enriched GO biological processes involved in response to oxidative stress and toxic substances for the Day 0 and Lactate-stressed Day 12 samples (Fig. 4c). Notably, eccDNA gene ratios were higher in the lactate-stressed samples compared to the Day 0 samples, despite the substantial different sizes of the gene lists. No significantly enriched terms were identified for the Control Day 12 samples (Fig. 4c).

### Characteristics of eccDNA biogenesis sites

**Figure 5:**
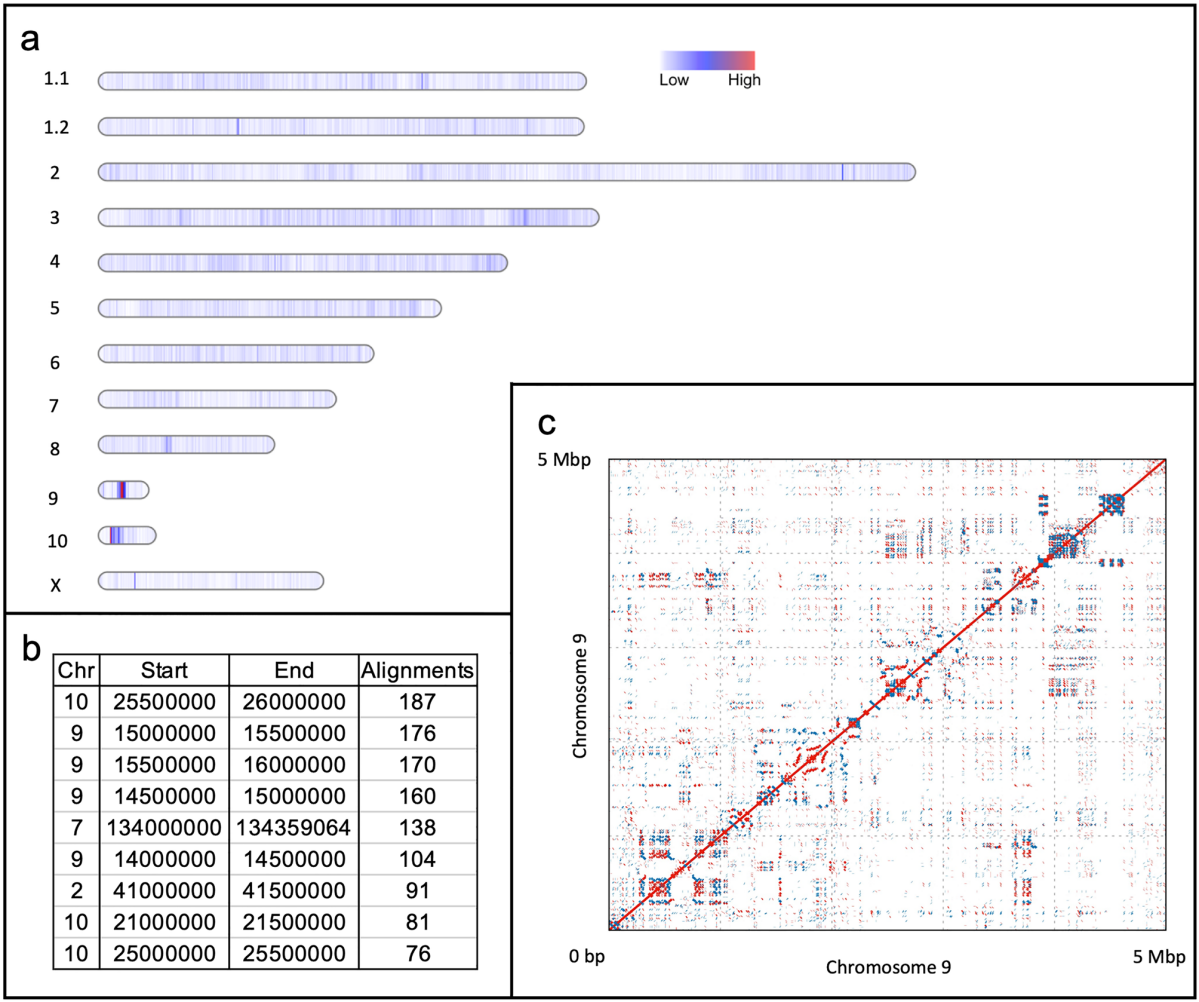
Observed eccDNA biogenesis sites and characteristics. (**a**) Chromosome-scale heatmap of eccDNA sequences observed for the Day 0 samples. Frequency of observed eccDNA is shown in color; low (white) to high (red). Figure made with minimap2 ^48^ and bedtools^47^. (**b**) 500 kbp windows with the highest eccDNA mapping frequencies (Top 10 from Day 0 only). (**c**) Self-alignment of chromosome 9 biogenesis hot spot. Red lines indicate a direct repeat; blue lines indicate an inverted repeat. Plot made using Mummer^65^.

There is very little knowledge regarding genomic origins of eccDNA as of this writing, thus to gain a better understanding of this process, linearized eccDNA sequences were aligned to the CHO PICRH reference assembly, binned into 4602 non-overlapping 500 kbp windows, and counted to identify the genomic distribution of biogenesis sites and possible hotspot regions. If an eccDNA is mapped to two windows, it is assigned to the leftmost window, however this occurrence is highly infrequent. EccDNA biogenesis sites were identified throughout the genome with several regions that had higher eccDNA mapping rates, areas considered to be hotspots. Interestingly, only 22 genomic windows (500 kb) out of the total 4602 500 kbp windows (< 0.5% of the genome) had no eccDNA mapped to these windows within the Day 0 samples (Supplementary Table S15). For the Day 0 samples, there were 44,402 unique eccDNAs that mapped to the reference genome (Fig. 5a). The mean number of eccDNA mapped to a window was 9.64 (standard deviation of 8.02). To identify windows with the highest frequency of eccDNA mapping, windows were assigned Z-scores based on eccDNA mapping frequency; 58 windows had a Z-score ≥ 2 corresponding to 26 or more instances of mapping, and six windows had ≥ 100 mapped eccDNA sequences. One window on chromosome 10 had the highest number of unique alignments with 187 eccDNA sequences. Chromosome 9 had a region spanning ~ 2 Mbp that harbored 610 eccDNAs (Fig. 5b). This region on chromosome 9 also contains 30 unnamed genes, seven of which are described as chromatin target of *Prmt1* protein-like, five are zinc-finger proteins, and one related to a growth inhibitor protein. A self-alignment of this region on chromosome 9 revealed a repetitive structure with dispersed direct and inverted repeats, tandem repetitive arrays, palindromic sequences, and regions with the potential for intramolecular recombination (Fig. 5c). Maps for the Control Day 12 and Lactate-stressed Day 12 biogenesis sites can be found in Supplementary Figs. S4 and S5 respectively, and summaries of biogenesis the frequencies can be found in Supplementary Tables S16 and S17.

The biogenesis analysis of the Day 12 samples identified similar patterns to the Day 0 samples. Control Day 12 samples had 14,457 unique alignments while Lactate-stressed Day 12 samples had 11,028 unique alignments. The mean biogenesis frequencies for Day 12 conditions were 3.14 and 2.39 for for Control and Lactate-stressed samples, respectively, with respective standard deviations of 3.25 and 2.4. Control Day 12 samples had 97 windows with Z-scores ≥ 2 while the Lactate-stressed Day 12 samples had 125 windows with a Z-score ≥ 2. Some variation in biogenesis frequency was observed between the three conditions; however, the 2 Mbp region on chromosome 9 and the 500 kbp window on chromosome 10 were ranked in the top 10 biogenesis sites for all three conditions. Detailed Information on biogenesis frequency and Z-scores for all conditions can be found in Supplementary Tables S15–S17.

### Identification of transcriptionally active eccDNA

**Figure 6:**
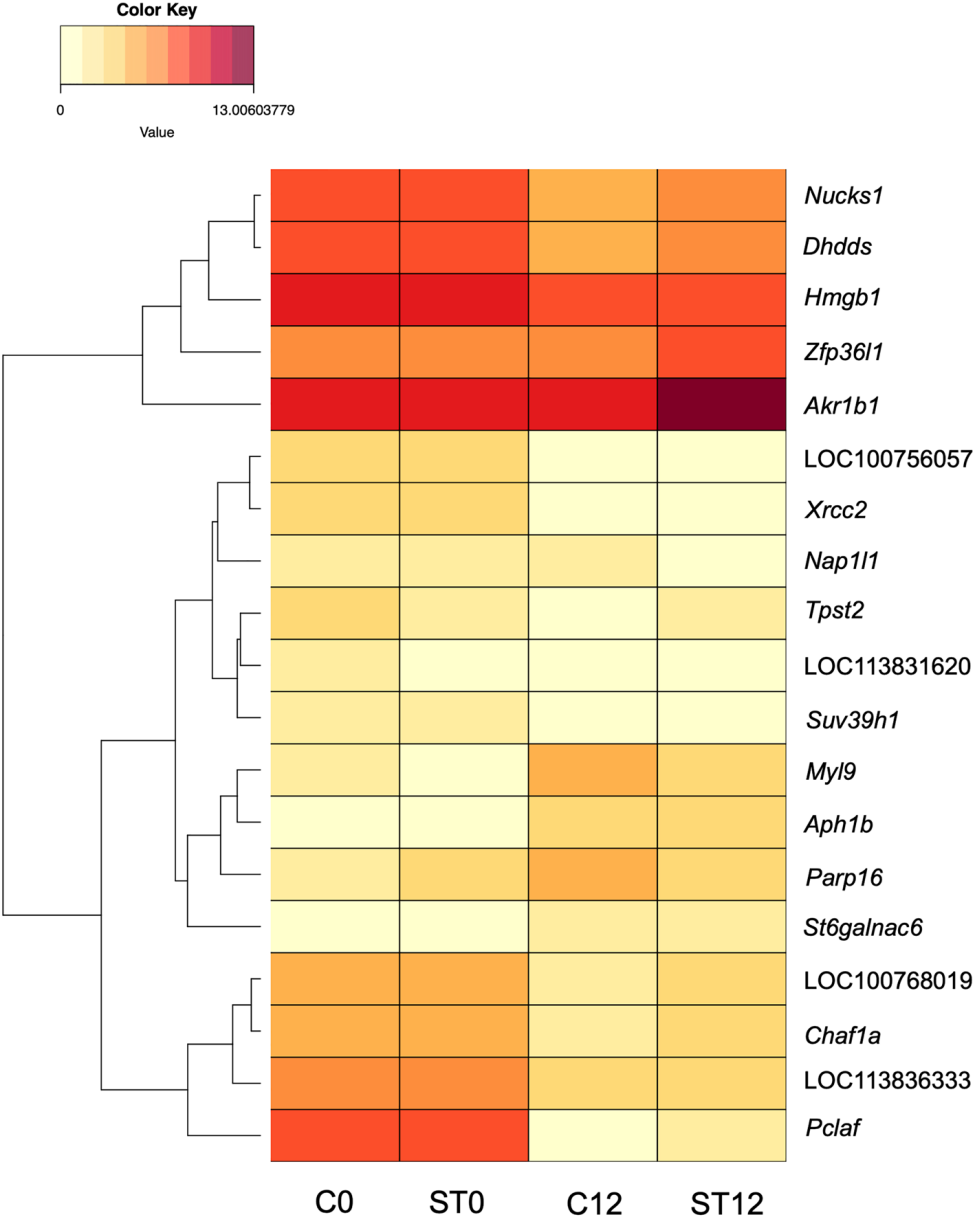
Heat map of differentially expressed genes from RNA-seq analysis that were only observed in one culture condition. Control is abbreviated as C and Lactate-stress is abbreviated as ST. Numbers in abbreviations are indicative of the Day samples were taken. Cells with light levels of shading indicate low levels of transcript abundance while darker cells correspond to higher levels of transcript abundance.

To identify eccDNA genes that may be transcriptionally active, RNA-seq data was intersected with eccDNA data. Observed eccDNA genes that were unique to the Day 0 samples (916 genes) and genes unique to Day 12 samples (448 genes), were intersected with genes found to have a ≤ − 2 or ≥ 2 log_2_ fold change respectively to correlate eccDNA gene loss or gain with corresponding transcriptome differences. Of the 916 genes only observed in Day 0 samples, 13 genes correlated with reduced transcript abundance. Of the 248 genes found only in Control Day 12 samples, 2 were correlated with increased expression; and for the 200 genes found in the Lactate-stressed Day 12 samples, 4 followed this pattern (Fig. 6). For context, 996 genes were found to be downregulated in one or both Day 12 conditions while 1002 were found to be upregulated in one or both Day 12 conditions. This implies that eccDNA had minimal impact in global gene expression shifts. The RNA-seq gene expression data for the 19 eccDNA genes is summarized in Supplementary Table S18 and global gene expression shifts are shown in Supplementary Table S19.

**Figure 7:**
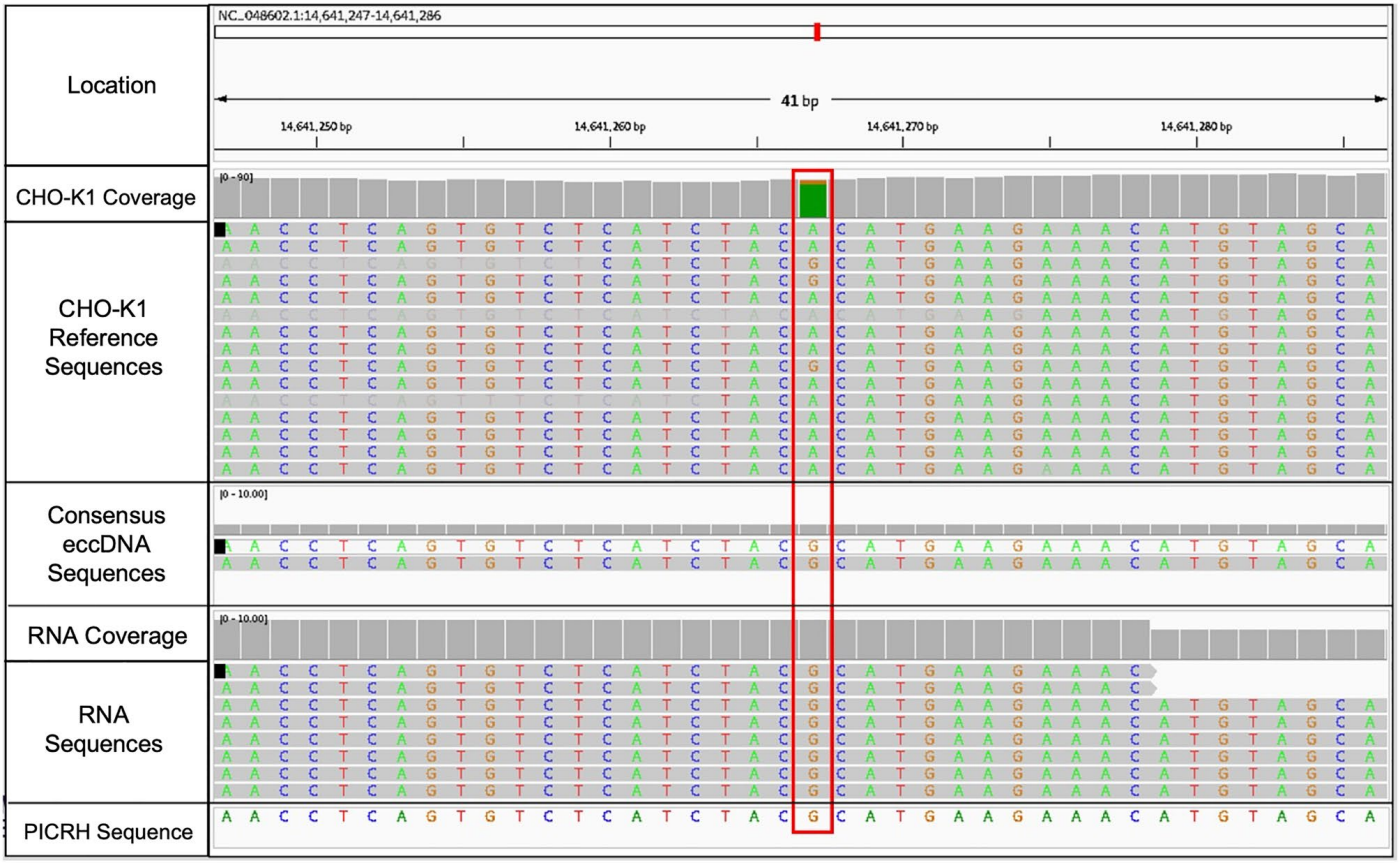
Example of transcripts with regions containing SNPs that may have originated from an eccDNA template on chromosome 9 at base 14,641,267. Rows from top to bottom are: location of the sequence, coverage of the CHO-K1 reference assembly, the CHO-K1 reference genome, consensus eccDNA sequence for the Lactate-stressed Day 12 samples, coverage of the RNA data, RNA transcripts for the Lactate-stressed Day 12 cultures, and the Chinese hamster PICRH reference genome. The height of the gray bars in the coverage row reflects the amount of coverage for each nucleotide. Colored bars in the coverage row reflect SNPs relative to the reference genome. Nucleotides are shown as: adenine (A)—green, thymine (T)—red, guanine (G)—orange, and cytosine (C)—blue.

It is likely that genes encoded on eccDNA are under alternative selective pressure compared to chromosomal genes. To assign RNA transcripts directly to eccDNA, the RNA transcripts and eccDNA were called for variants against both the CHO-K1^66^ and the Chinese hamster PICRH^67^ reference genomes to identify single-nucleotide polymorphisms (SNPs) or insertions/deletions (INDELs) specific to eccDNA. For example, RNA transcripts containing a SNP relative to the CHO-K1 reference genome that is also within the consensus eccDNA sequence may have originated from the eccDNA template. Using this approach, homozygous SNPs were identified on an eccDNA and corresponding transcripts on chromosome 9 at base 14,641,267. For the CHO-K1 reference genome, 89% of the reads (63) at this location are adenine (A), with the remaining reads (8) being guanine (G). The consensus eccDNA and RNA transcripts at this locus are both guanine (G) in 100% of reads (Fig. 7).

## Discussion

Genome instability among CHO cell lines is a major contributor to declining productivity and product quality as cultures age; long-term cultured (LTC) cells have also been shown to have altered carbon metabolism due to genome instability^2,4^. The altered metabolism of LTC cells implies that genome instability has broader impacts beyond expression and glycosylation of recombinant protein products. Genome instability occurs through a variety of mechanisms such as chromatin condensation^68^, DNA methylation^69^, and variant accumulation^7^. Variant accumulation is often accelerated when DNA repair and or recombination mechanisms are compromised, which can be observed via biomarkers such as microsatellite instability^7^. Cell cultures are highly dynamic environments that constantly change in sometimes undesirable ways. Multiple factors, such as nutrient depletion, increased cell densities, and waste product accumulation, create stress within a culture that may elicit a stress response within the cells^70^. Cells have innate signaling and regulatory mechanisms that govern gene expression and, ultimately, the phenotype of the cell population. The cascades of these mechanisms that lead to adaptation have been understood to be encoded and maintained within the main chromatin body. Recent evidence has shown that eccDNA can harbor and express genes, influence gene expression of chromosomally encoded genes, and rapidly respond to cellular stress^28,71,72^. EccDNA are poorly understood in many systems and may function in the genetic coordination of traits in CHO under stress and homeostatic conditions.

Lactate was chosen as the stress in this study because it is a common waste metabolite; reducing or eliminating lactate is an intense area of research^16,17,70,73–75^. Elevated levels of lactate contribute to culture acidification; in controlled systems, such as the ambr^®^250, this can cause the system to add excessive base and/or increase the pCO_2_ level, which can increase the osmotic pressure on the cells and retard growth^6,76,77^. Furthermore, lactate has also been shown to stunt cell growth and limit cell-specific productivity ^78,79^. Previous work showed that lactate becomes detrimental at approximately 20 mM, and culture termination will occur in concentrations exceeding 40 mM^80^. The lactate stress was added incrementally in this study to avoid an overstressed environment while creating sufficient stress on the cultures. Preliminary shake flask experiments demonstrated that 10 mM doses of lactate allowed for better growth than a single 30 mM addition^81^. Further, as the osmolarity of the Control and Lactate-stressed cultures were not different, the slightly higher volume addition (< 2.2%) to supplement the lactate was considered an insignificant effector (*p* < 0.05). The reduced VCD and cell specific productivities of the stressed cultures show that a sufficient stress was achieved, while the tightly matched viability of all cultures demonstrates that the stressed cultures were not overwhelmed. Thus, the lactate-stress was the major effector, as well as culture time.

Capture and sequencing of eccDNA is a relatively new area of molecular biology that has been made more accessible by the rapid evolution of single-molecule sequencing technology such as Pacific Biosciences high fidelity (HiFi) reads. Circular DNA enrichment sequencing (CIDER-Seq) is an approach that circumvents the need for complex molecular protocols and computationally intensive analysis^43^. While the CIDER-Seq makes the identification of circular DNAs more robust, a caveat to the procedure is that it is not quantitative due to the uncontrolled enrichment of eccDNA via rolling circle amplification (RCA). It is also critical to note that this technique is biased toward smaller sequences as these sequences are capable of much faster replication and hence accumulate more rapidly than larger sequences. CIDER-Seq is also limited to the read length offered by the sequencing instrument, but as sequencing technologies improve, it is anticipated that a parallel improvement in resolution of these elements will occur. It is possible that longer eccDNAs exist, but were missed due to these current biases. Other methods that do not rely on Phi-29 amplification can be employed to estimate eccDNA abundance; however, these methods are much more costly and require a prohibitive amount of starting material. These cost and material requirements make characterizing the full distribution of discreet eccDNA size and abundance prohibitive for CHO cell culture experiments on this scale. Despite the limitations associated with the CIDER-Seq methodology, it still yields high quality data for sequences between approximately 20 and 25,000 bps.

Analysis of repetitive regions shows that the distribution of repeat structures within eccDNAs observed in this study were relatively equal across each condition. The similarity of repeat motifs across experimental groups suggests that biogenesis of eccDNAs due to repeat overlapping remains consistent in CHO cells when grown in both control and lactate-stressed conditions. The most identified repetitive element observed across all conditions was LINE1 (long interspaced nuclear element). In humans, LINE1 makes up approximately 17% of the genome^82^. While many LINE1s are transcriptionally silent in humans, some are capable of retro transcription, which can cause disruptions via insertion, deletion, or rearrangement^83^. Another notable repeat motif, SINEs (short interspaced nuclear element), was also observed in relative abundance on eccDNA for all three conditions. SINEs are another type of retrotransposon that make up about 13% of the mammalian genome^84^. Structurally, SINEs have a conserved sequence structure as these transposable elements originate from tRNA sequences^85^. A relatively large portion of observed eccDNA in this study (8.98%) carried one or more tRNA motifs. EccDNA harboring tRNA has been previously described in *Arabadopsis*^86^. It is speculated that maintaining tRNA genes extrachromosomally may aid in stress response by facilitating rapid or high protein turnover required by a dynamic transcriptome load^87^. Other work has established that tRNA abundance is selectively modulated under stress conditions to regulate protein synthesis in yeast^88^. This could suggest an additional function of eccDNA within modulating protein production beyond gene expression.

When eccDNA sequences were annotated for genes, an average of ~ 3.35% were found to have one or more genes. Again, because the CIDER-Seq protocol is not quantitative, this is not indicative of the abundance of coding eccDNA, but rather a representation of eccDNA with predicted gene sequences. Most of the identified genes were only observed in one of the three conditions. This implies that gene content is highly dynamic across a 12-day fed-batch culture and between stressed and control conditions. A functional enrichment analysis using gene ontology identified significantly enriched GO terms among each of the three culture conditions, many of which were linked to ribosomal assembly, cytoplasmic translation, and ncRNA processing. This enrichment could be influenced by the host cell line (CHO K-1) and/or the cell line development process. While we cannot comment on eccDNA content of the CHO K-1 host, it is highly likely, if not certain, that eccDNA is present due to the ubiquitous nature of eccDNA in normal^27,29^, disease^31,89^, and stressed states^28^.

Identifying multiple GO terms linked to translation could be attributed to selection of a clone with a high-producing phenotype during cell line selection as this is desirable for biomanufacturing. These eccDNA genes could be widely dispersed through cells in culture if present in the original clone; however, without selective pressure, these genes could be lost over time, which would likely result in reduced productivity. Clones with high-producing phenotypes have been observed to lose the desired phenotype over time^90^. While there are multiple factors that could contribute to this, such as transgene exclusion and variant accumulation^2^, eccDNA-mediated loss of productivity has not been studied in recombinant CHO cell lines. Further, the presence of *Dhfr* in all three conditions could be indicative of attempted transgene exclusion as *Dhfr* is the selectable marker used for the CHO K-1 cell line^64^. More ontology terms were significantly enriched in the Day 0 samples, however, this was likely due to the pooling of all four bioreactors for the Day 0 gene list as opposed to two bioreactors each for the Control and Lactate-stressed Day 12 gene lists. Yet, the Day 0 gene list had more than twice the number of genes compared to the Day 12 lists (1622 genes in Day 0 vs. 486 for Control Day 12 and 451 for Lactate-stressed Day 12). While the Lactate-stressed Day 12 and Control Day 12 had some variation in enriched GO biological process terms, none of the terms observed in the Lactate-stressed Day 12 group were indicative of a stress response, but rather translation, ncRNA processing, and ribosome assembly; significantly enriched terms observed in the Control Day 12 genes also pertained to protein production. Maintaining genes related to protein production on eccDNA likely aid the cells in facilitating protein turnover.

Biochemical pathway analysis of the observed genes that are associated with eccDNA in humans from the literature showed a significant enrichment in multiple cancer pathways. Linkages between eccDNA and cancer are clear as both eccDNA biogenesis, and cancer progression often rely on compromised DNA repair and or recombination mechanisms^29,32,34^. Additionally, some of these genes, such as *Poli* (error-prone polymerase involved in DNA repair), *Rac1* (cell growth regulator), and *Palb2* (tumor suppressor) were observed on eccDNA in CHO cells (Fig. 4b). Overexpression of these genes could accelerate cell division, increase eccDNA biogenesis or recombination, and hasten the onset of cell line instability. Ontology of genome instability linked orthologs for the genes observed on eccDNA in CHO cells showed a notable increase in genes related to oxidative and toxic substance stress response in the Lactate-stressed Day 12 samples, which were not observed for the Control Day 12 samples. Furthermore, the fraction of cancer genes increased in the Lactate-stressed Day 12 samples compared to the Day 0 samples despite having a much smaller number of eccDNA genes observed.

EccDNA biogenesis has been shown to occur through multiple error-prone pathways such as non-homologous end joining (NHEJ), double-strand break repair, chromothripsis, and transcription; errors in DNA repair pathways are among the most prominent biogenesis mechanisms^33,41,91,92^. Regions of the genome enriched with tandem repeats and other repetitive motifs have been observed to be more susceptible to eccDNA formation^93,94^. Due to the varied nature of eccDNA biogenesis mechanisms, eccDNA in humans appear to arise almost ubiquitously from the genome^72^. This allows some eccDNA to carry other functional sequences, such as autonomous replication sequences that enable gene copy number amplification and eccDNA permeation^28,95^. Centromeres have yet to be identified on eccDNA^31,32,89^. Thus, eccDNAs typically display uneven segregation between daughter cells, which can increase population heterogeneity. When mapped to the genome, eccDNA biogenesis was found to occur globally throughout the genome; however, a 2 Mbp region near the center of chromosome 9 was found to have the highest frequency of biogenesis, likely due to the repetitive sequence structure of the region as shown in the chromosome self-alignment (Fig. 5c). It was also observed that 3.35% of observed eccDNA contained one or more genes or gene fragments. This is an overrepresentation of genes when compared to humans, as less than 2% of the human genome consists of coding genes^82^. EccDNA being biased toward coding regions of the genome supports previous work published by Hull, which correlated elevated levels of gene transcription with higher eccDNA abundance in yeast^91^.

While genes may be amplified in eccDNA, additional regions, such as promoter and transcription factor binding sites, are required for transcription may be excluded or mutated. There is ambiguity when attempting to assign a transcript to an eccDNA or chromosomal template. The most direct approach to identifying eccDNA-derived transcripts is to leverage eccDNA-specific variants relative to the chromosome-encoded gene. It can be assumed that an eccDNA encoded gene is under alternative selective pressures than chromosomal genes, hence eccDNA-encoded genes may accumulate variants at different rates. However, focal amplifications in the form of eccDNA may contain an exact copy of a nuclear gene, making it impossible to know which copy is functional. Mapping variant transcripts to the respective template is a straightforward way to identify sequence origin; however, this would not reflect transcripts from high-fidelity eccDNA that exactly matches its genomic template. In addition to having proper transcription machinery, eccDNA need a replication origin to permeate through the population after selection. Yet, a single sequence does not need to have replication origins, promoters, and gene bodies upon biogenesis to permeate through the population, as recombination between eccDNA can allow for the accumulation of functional elements. While the timespan in this experiment was short (12 days), recombination events likely occurred in these sequences, but would be more prominent in longer cell cultures, such as perfusion.

Of the thirteen genes only identified in Day 0 eccDNAs that were found to be downregulated by Day 12, one is involved in maintaining genome stability (*Nap1l1*), three facilitate DNA repair (*Nucks1, Pclaf,* and *Xrcc2*) and three maintain or regulate chromatin structure (*Suv39h1, Chaf1a,* and *Nap1l1).* Two genes only observed on the Control Day 12 eccDNAs were observed to have increased transcription, while four genes were observed on the Lactate-stressed Day 12 eccDNAs that were upregulated. The two upregulated genes observed on the Control Day 12 eccDNAs included two signal transduction genes (*Myl9, Parp16*). The four upregulated genes on the Lactate-stressed Day 12 eccDNAs include *St6galnac6,* a cell surface receptor gene*, Zfp36l1,* a zinc finger protein*, Aph1b,* a transmembrane protein that is part of the gamma-secretase complex^96^, and *Akr1b1*, an aldo–keto reductase that catalyzes NADPH-reduction of carbonyl-compounds into alcohols^96^. Overexpression of *Akr1b1* has been observed in multiple cancer types and is thought to increase Warburg effects by triggering the AKT/mTOR signaling pathway^97^. *Akr1b1* overexpression in the Lactate-stressed Day 12 cultures could have contributed to the elevated lactate production and subsequent accumulation observed starting on Day 8.

## Conclusion

This work has demonstrated that the eccDNA gene content within CHO cells is highly dynamic, even across the relatively short time span of a fed-batch culture. While tightly controlled bioreactor systems, such as the ambr^®^250, are lauded for the tight level of culture control, internal genetic elements can still drive heterogeneity that leads to phenotypic drift. These issues may be difficult to address with process engineering and likely present a new challenge in cell line development efforts to curb genetic heterogeneity. EccDNAs in CHO cells may bias the clone selection process by harboring beneficial genes for protein expression, modification, and secretion; yet, during production, conditions may allow phenotypic drift through amplification of genes responsible for cancer phenotypes or loss of beneficial genes that are unevenly segregated or lost in cell division. Furthermore, this work highlights the importance of eccDNA microevolution due to environmental disturbances, such as waste metabolite accumulation. Thus, eccDNA may be considered as new targets for CHO cell line improvement and genetic process control.

## Supplementary Information

Supplementary Figures.

Supplementary Tables.

Supplementary Tables.

## Data Availability

All sequence data generated and/or analyzed in this study are available in the NCBI sequence read archive under BioProject: PRJNA896947 Submission ID: SUB12241330. All other data generated or analyzed during this study are included in the published article and its supplementary information files.
