# Supplementary material for: Microevolutionary dynamics of eccDNA in Chinese hamster ovary cells grown in fed-batch cultures under control and lactate-stressed conditions
Source: Sci Rep. 2023 Jan 21;13:1200. doi: 10.1038/s41598-023-27962-0 (PMC9862248; doi:10.1038/s41598-023-27962-0)
Supplement: Supplementary file 1 — Supplementary Figures. [file 41598_2023_27962_MOESM1_ESM.pdf]

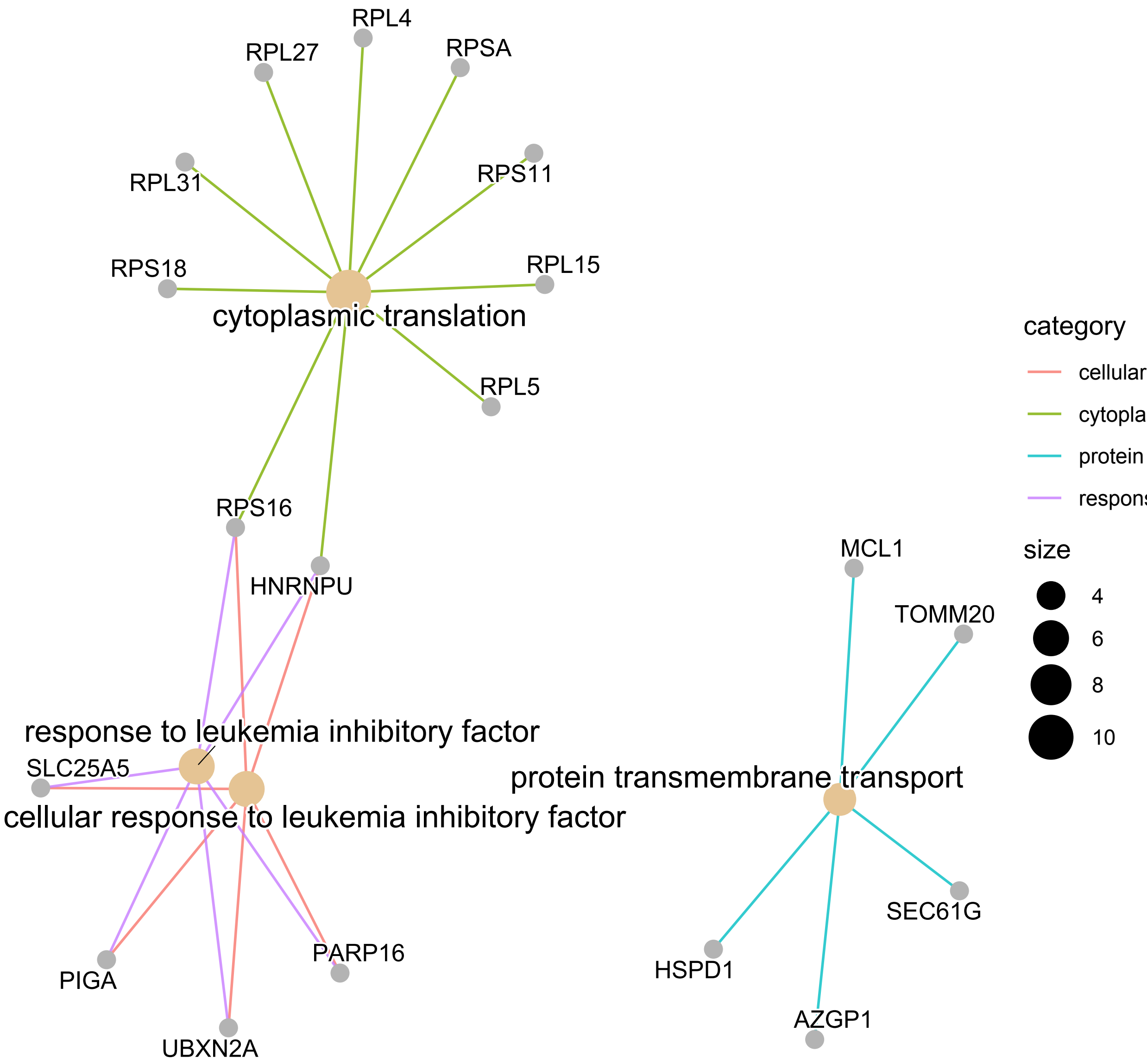

Figure S1: Control Day 12 BP. Network diagram of significantly enriched GO biological process terms for the human orthologs of Chinese hamster genes detected in Control Day 12 samples. The small gray nodes show individual genes and larger beige nodes indicate GO terms. The size of the beige nodes is proportional to the number of genes with that GO term and the colored lines indicate the GO category for which a gene belongs to.

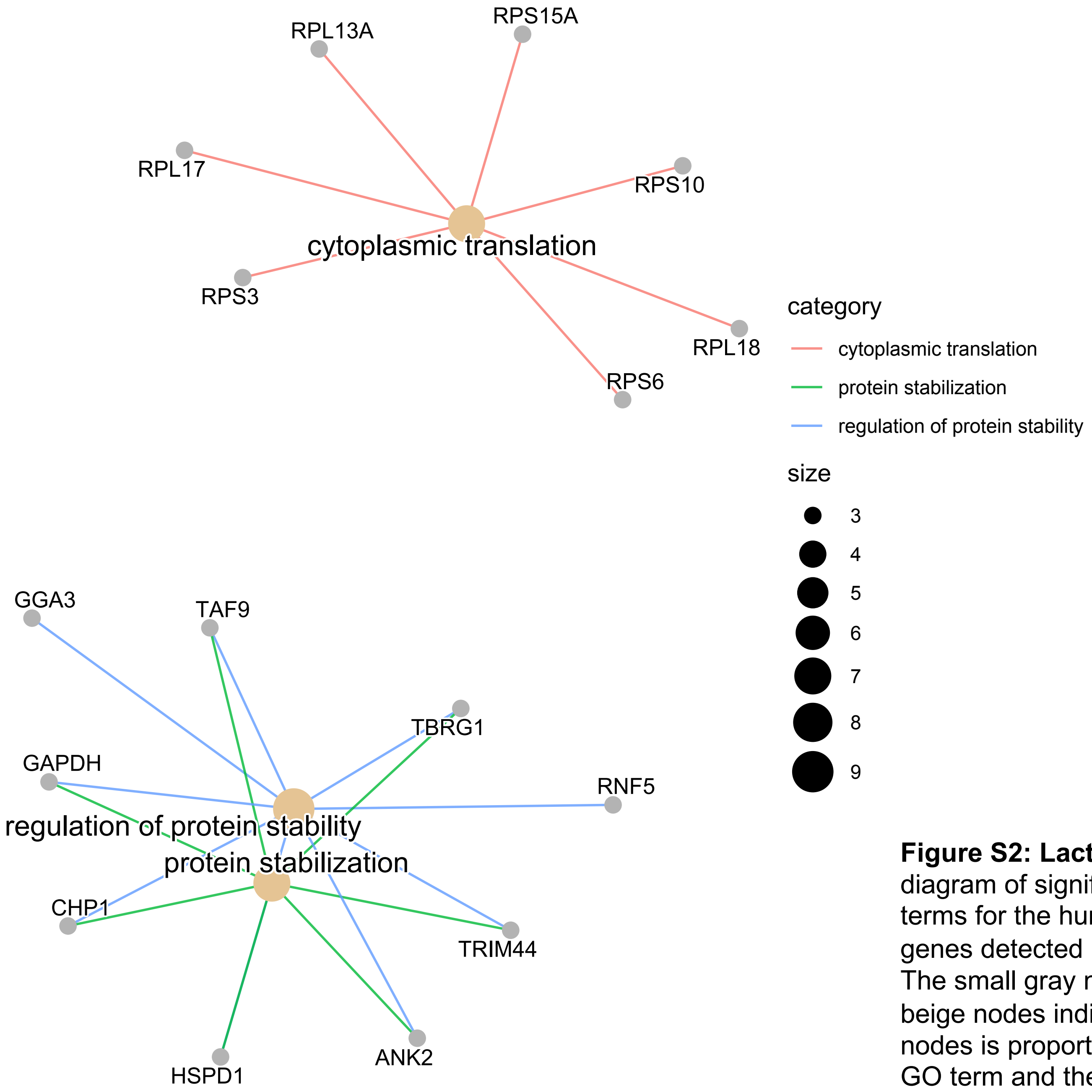

Figure S2: Lactate-stressed Day 12 BP. Network diagram of significantly enriched GO biological process terms for the human orthologs of Chinese hamster genes detected in Lactate-stressed Day 12 samples. The small gray nodes show individual genes and larger beige nodes indicate GO terms. The size of the beige nodes is proportional to the number of genes with that GO term and the colored lines indicate the GO category for which a gene belongs to.

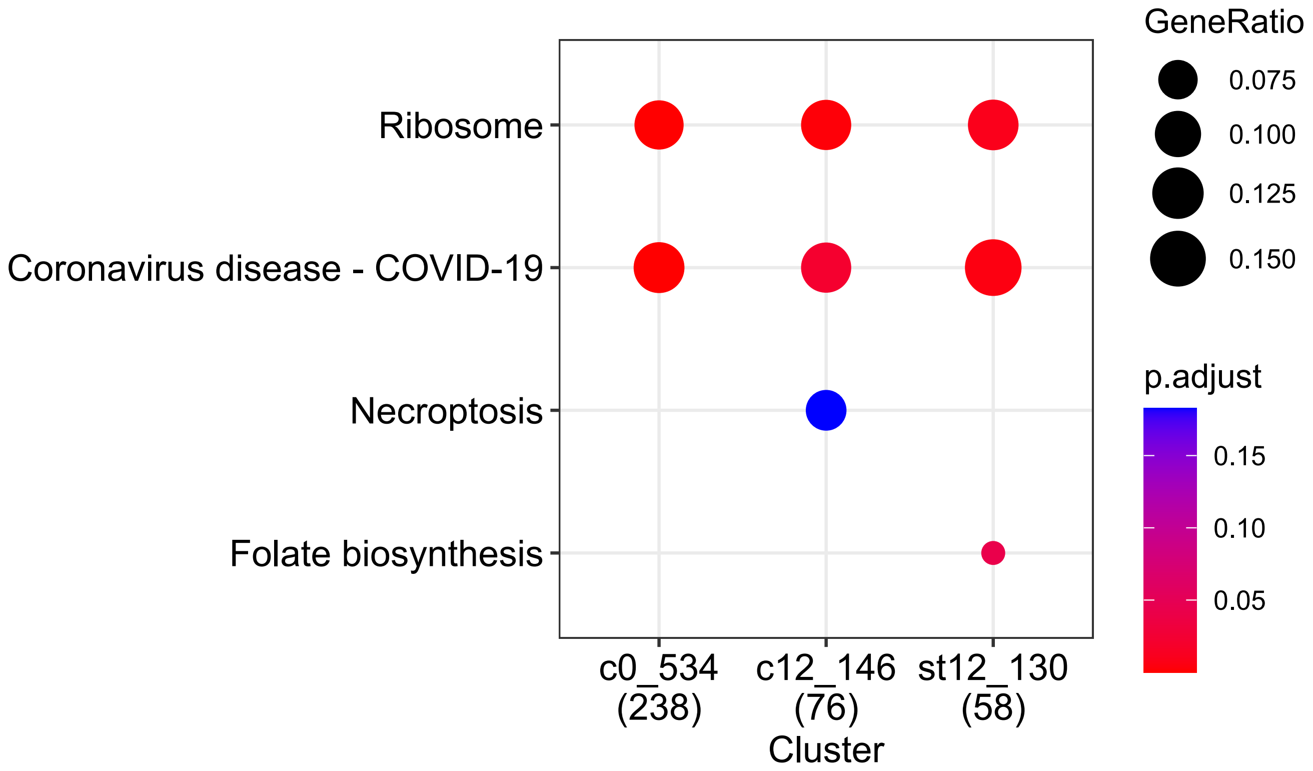

Figure S3: KEGG Pathway analysis of observed eccDNA genes. Node size is proportional to the number of genes found in the pathway, while the node color indicates the pathway's statistical significance.

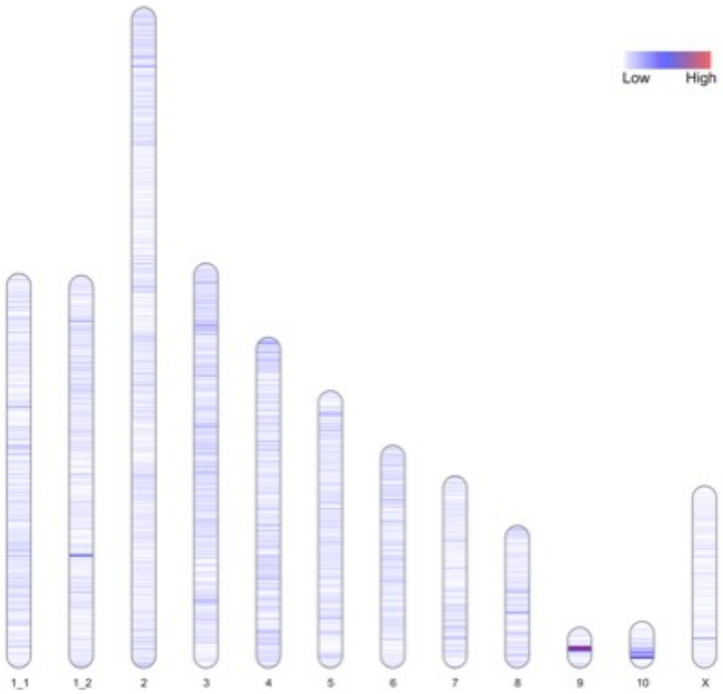

Figure S4: Control Day 12 biogenesis map. Chromosome-scale heatmap of eccDNA sequences observed for the Control Day 12 samples. Frequency of observed eccDNA is shown in color; low (white) to high (red).

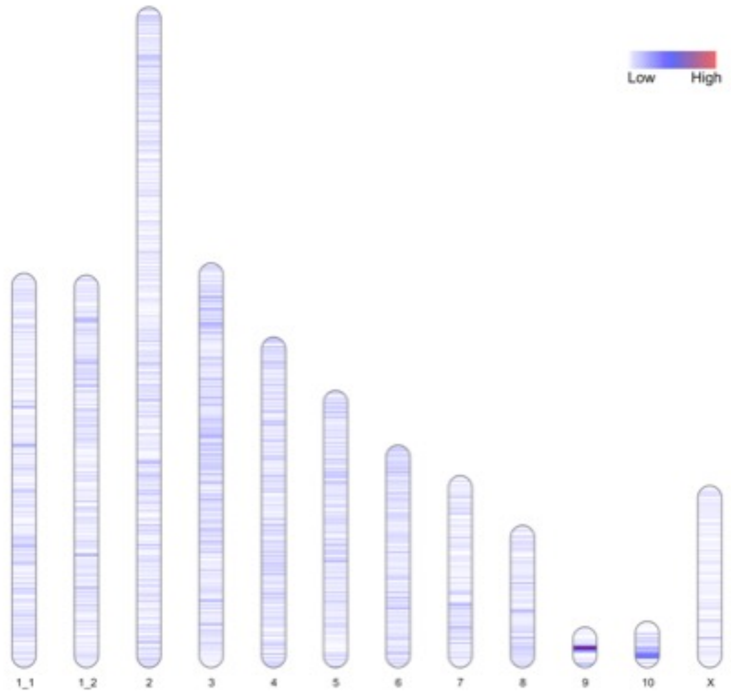

Figure S5: Lactate-stressed Day 12 biogenesis map. Chromosome-scale heatmap of eccDNA sequences observed for the Lactate-stressed Day 12 samples. Frequency of observed eccDNA is shown in color; low (white) to high (red).
